# Performance of a CO_2_ Impedimetric Sensor Prototype for Air Quality Monitoring

**DOI:** 10.3390/s110505047

**Published:** 2011-05-05

**Authors:** Gemma García Mandayo, Jaime Herrán, Irene Castro-Hurtado, Enrique Castaño

**Affiliations:** 1 CEIT and Tecnun, University of Navarra, Manuel de Lardizábal 15, 20018 San Sebastian, Spain; E-Mails: ichurtado@ceit.es (I.C.-H.); ecastano@ceit.es (E.C.); 2 CIDETEC, P. Miramón 196, 20009 San Sebastián, Spain; E-Mail: jherran@cidetec.es

**Keywords:** carbon dioxide, air quality, thin film, BaTiO_3_-CuO

## Abstract

Carbon dioxide detection is a relevant issue in many fields, and this work focuses on the use of a BaTiO_3_-CuO sputtered thin film layer in a gas sensor prototype for air quality measurements. For this, a double side sensor was fabricated, with a Pt heater on one side and the sensing layer over the electrodes on the other side. The uniformity of the temperature on the sensing layer was tested and further tests to check its sensing performance were carried out. Humidity influence in the detection was found to be almost negligible within the usual range in air quality measurements and repeatability tests show satisfactory results for air quality control purposes.

## 1. Introduction

Carbon dioxide sensors are devices in high demand for a wide range of applications, ranging from intelligent food packaging, greenhouse control, to air quality monitoring. Focusing on air quality, CO_2_ concentration in a room gives information about the necessity of ventilating it. In order to quantify that need, the American Society of Heating, Refrigeration, and Air-conditioning Engineers (ASHRAE) recommends a ventilation rate that leads to an indoor concentration of 870 ppm, taking a base concentration of 350 ppm outside the building. It can be stated that CO_2_ concentrations in office buildings typically range from 350 to 2,500 ppm. Thus, the amount of CO_2_ indoors can be used to rationalize the use of the HVAC (Heat, Ventilation and Air Conditioning) systems. If the increase of the energy costs nowadays is taken into account, any action aiming at reducing energy consumption is a relevant issue. The carbon dioxide concentration measurement is currently being used by some HVAC product manufacturers. One of the devices used for that purpose is the Carbocap, manufactured by Vaisala. As in the case of the Carbocap, most of the CO_2_ sensors available in the market are based on the infrared detection (IR). Recently, some NDIR miniaturized detectors for CO_2_ were commercialized by other companies (Alphasense, Dynament). However, they still remain expensive for certain applications. Electrochemical sensors (Figaro, Alphasense, Hanwei Electronics) are the cheapest option available at the moment. Table 1 shows some of the models available, their detection range and their approximate price.

Semiconductor resistive, capacitive or impedimetric devices are also a low-cost good choice for gas sensors. BaTiO_3_-CuO was used in the past by several authors for carbon dioxide detection in the form of pellets or thick films [4–8]. The authors obtained better results than other groups by using a thin-film layer of BaTiO_3_-CuO [9–12]. This layer can be both deposited onto alumina and silicon, because the adherence of BaTiO_3_-CuO thin layers (below 400 nm) to both silicon and alumina substrates is good, but nowadays, some manufacturers are still reluctant to the use of silicon as a substrate, therefore, the research effort described in this work is focused on the development of a cheap CO_2_ sensing device on an alumina substrate. A preindustrial prototype that uses impedimetric measurements in a solid state sensor based on a mixed binary oxide (BaTiO_3_-CuO) was fabricated and characterized.

## 2. Experimental

**Figure 1.**
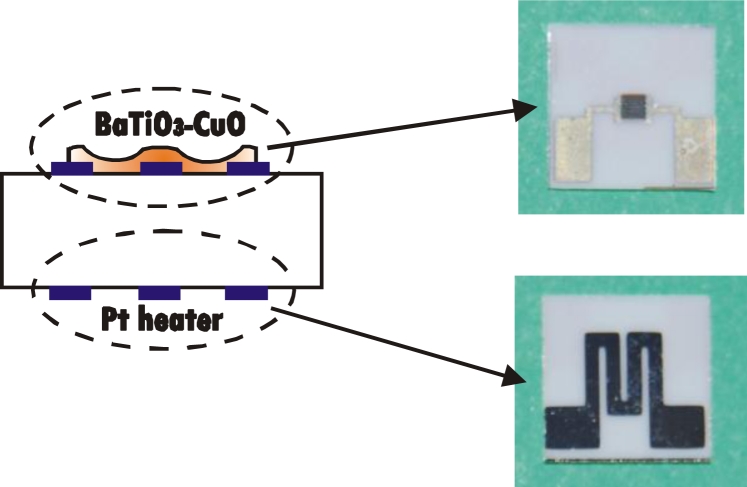
Sensor cross-section and photograph of the final chip (top and back side).

A double-side impedimetric gas sensing device was fabricated on a 0.5 mm thick alumina substrate giving the 6.25 × 6.25 mm^2^ chip shown in the diagram and photographs of Figure 1. On the back side there is a platinum heating resistor that has been optimized to get a uniform temperature on the sensing area and to control the operating temperature in an accurate way. On the top side, platinum interdigitated electrodes are deposited by DC sputtering in order to perform electrical measurements of the sensing layer of 1 × 1 mm^2^. The BaTiO_3_-CuO films are deposited on the electrodes by RF sputtering and then annealed in oxygen at 700 °C for 3 hours, as explained in [9]. The thickness of the samples is measured with a profiler P-16+™ of KLA Tencor.

The electrical characterization of the sensors is performed inside a sealed stainless steel chamber, where the target atmosphere is obtained by means of a mixing system consisting of mass flow controllers (MFCs) from Bronkhorst Hi-Tech controlled by a PC. A Dynamic Data Exchange communication is established between the computer and the MFCs to operate them by Labview^©^.

Impedance measurements are performed at a fixed frequency and at a temperature of 300 °C. The test frequency is 100 Hz, because this value is within the range of frequencies that give maximum response [9] and the temperature is the one that gives the best combination of response magnitude and response time [10]. The samples are tested under a fixed flow of 400 sccm, and stabilised at the test temperature under a flow of clean air at 40% relative humidity.

**Figure 2.**
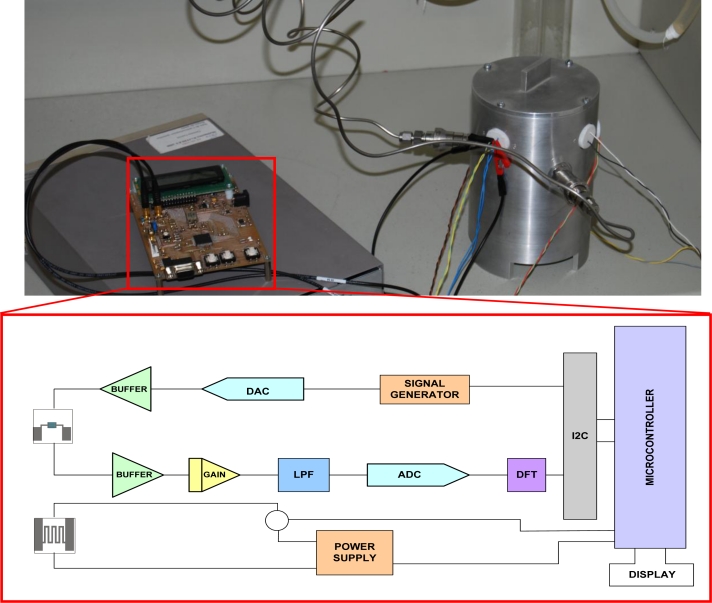
Signal conditioning circuit in test-bench and functional block diagram.

The electrical characterization is performed two different ways: the first impedance measurements shown are performed using a Lock-In amplifier (Model 7265, Signal Recovery), to check the repeatability of the prototype results in relation to the previously fabricated samples when using the same measuring system. The subsequent tests are performed with a signal conditioning circuit designed to control the temperature of the sample by applying constant power, and also to measure the impedance changes in the prototype (Figure 2). The system uses a Motorola MC68H900AZ60A microprocessor. The conditioning circuit is connected to the computer through a RS-232 connection in order to record the data measured by the circuit.

## 3. Results and Discussion

### 3.1. Heater Characterization

**Figure 3.**
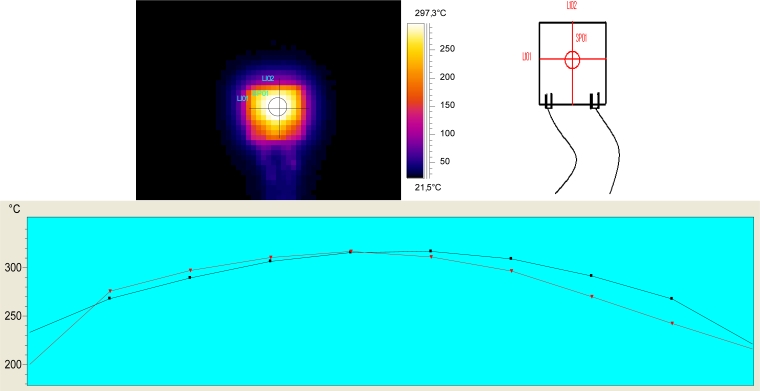
Temperature distribution on the surface of the film (top-left side) and temperature distribution (bottom) on the lines marked in the top-right chip scheme.

The aim of the heater is to provide a uniform temperature distribution all over the sensing film. The chips have been characterized by means of a thermographic camera to check the uniformity and the power consumption required to get the working temperature. The results, which can be seen in Figure 3, show that the distribution on the 1 × 1 mm^2^ square area in the center of the chip is indeed homogeneous.

Four heater prototypes were tested at different and the power consumption has been measured. The thin-film platinum heater designed shows a linear behavior in the graph power *vs*. temperature up to 500 °C. The power required for the working temperature of the samples tested in this work (300 °C) is 2 W.

### 3.2. Dynamic Response and Sensitivity of the Sensors

The first measurements were performed to check the reproducibility of the sensor response when compared to the previously published results measured on lab prototypes with external heating. The sensor response was measured within the range of 500 to 5,000 ppm. The test was performed introducing carbon dioxide pulses of the corresponding concentration for 30 minutes in the test chamber, followed by 30 minutes in clean air.

**Figure 4.**
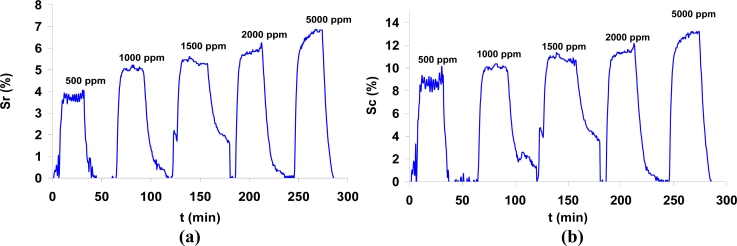
Dynamic response to carbon dioxide at a concentration of 500 to 5,000 ppm, measured at 300 °C and 40% RH. **(a)** Resistance change. **(b)** Capacitance change.

An example of the dynamic response of the samples can be seen in Figure 4. In this case the impedance variation was plotted both as the resistance variation and as the capacitance variation of the sample. As Figure 4 shows, resistance increases and capacitance decreases as the carbon dioxide concentration gets higher. The response and recovery times are 3 and 5 minutes, respectively, measured between the 10% and the 90% of the maximum response (t_10_ and t_90_). It must be taken into account that the volume of the test chamber is about 1 liter, so it takes more than 2 minutes to fill it with the test gas. Although the velocity of response measured this way is not high, the sensor is fast enough for indoor air quality assessment, because the response time in real conditions will certainly be shorter that the one reported here. These results are in line with the ones obtained in previous works [11], where the time of response was a function of the thickness of the sample. The thinnest sample measured in that case had a layer of 125 nm of BaTiO_3_-CuO, and it was the fastest one. Below that thickness, the impedance was very high for the signal conditioning circuit and it made the measurement difficult. Taking into account that the present prototypes show a thickness of 150 nm, the response rate agrees with the authors’ former works [11].

**Figure 5.**
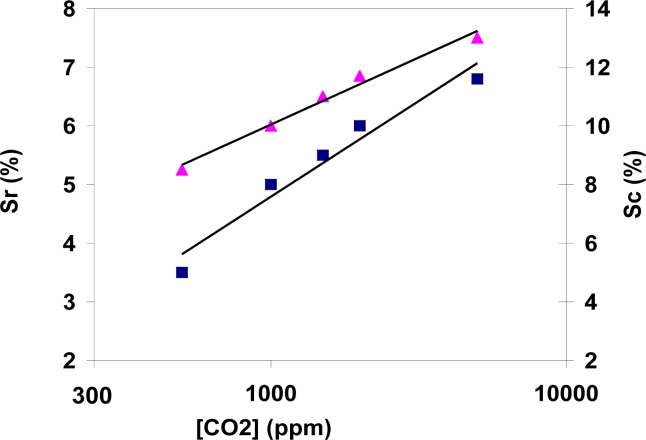
Sensitivity to carbon dioxide within a range 500 to 5,000 ppm, measured at 300 °C and 40% RH, in terms of resistance change (Sr) and capacitance change (Sc).

As it was also stated before [11], a linear behavior is observed only between 500 and 2,000 ppm, and the saturation of the sensor response happens above 5,000 ppm. Therefore, the response between 500 and 5,000 ppm can be deduced from the logarithmic plot of the sensitivity shown in Figure 5. This confirms the suitability of the sensor for air quality measurements. The response (S) of the sensors is measured in terms of capacitance (C), resistance (R) or impedance (Z), as shown in Equation (1), where X_0_ is the electrical parameter measured in clean air. In the case of capacitance, as it decreases when carbon dioxide concentration increases, the numerator of the equation changes to (X_0_–X).

(1) $${\text{S}}_{\text{x}}\;=\;\frac{\text{X}\;-\;{\text{X}}_{0}}{{\text{X}}_{0}}\cdot 100$$

### 3.3. Humidity Influence on the Sensor Response

**Figure 6.**
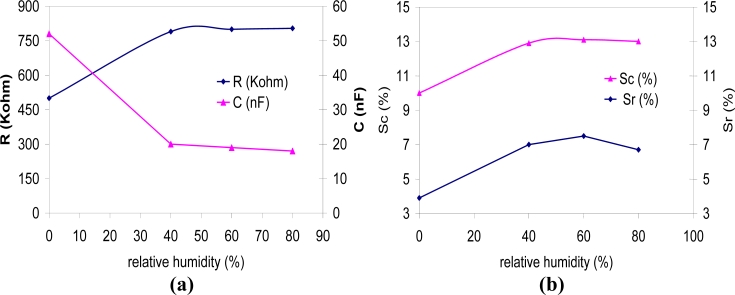
**(a)** Change in the resistance and capacitance baseline with relative humidity. **(b)** Change in the sensor response to 5,000 ppm (in terms of resistance Sr and in terms of capacitance Sc) with relative humidity.

The influence of relative humidity in the sensor performance is relevant in any air quality measurements. Therefore, the importance of this parameter was studied up to a maximum of 80% relative humidity. First, the changes in the baseline of the sensors resistance and capacitance were recorded, as shown in Figure 6(a). As it can be seen, there is a noticeable variation in the value of R and C between the measurements without humidity and the measurements with humidity, but within the range measured (40 to 80% RH), the impedance value remains almost constant.

The change in the sensor response to 5,000 ppm CO_2_ was also measured, as shown in Figure 6(b). Firstly, it can be observed that the response of the sensor increases when it operates under humidity conditions. Secondly, it can be stated that the influence of humidity in the range between 40 and 80% relative humidity is almost negligible. This feature is also appropriate for an air quality device, which would work within those humidity values.

As shown, sensitivity of BaTiO_3_-CuO is enhanced in the presence of water. According to the research carried out by Ostrick [13,14], carbon dioxide detection needs the presence of carbonate specimens [15–18], which was already demonstrated by DRIFT experiments for the BaTiO_3_-CuO thin films [12]. For temperature ranges over 200 °C sensitivity due to the carbonate specimen BaCO_3_ is activated by oxygen. As humid air contains a greater oxygen amount than dry air, this would explain the sensitivity enhancement measured in humid air. The reactions involved are shown below:

$$\begin{array}{l} {\text{CO}}_{2(\text{gas})}\;+\;\frac{1}{2}{\text{O}}_{2(\text{gas})}\;+\;2{\text{e}}_{\left(\text{metal}\right)}^{-}\;↔\;{\text{CO}}_{3(\text{material})}^{2-}\\ \begin{array}{c} {\text{CO}}_{3(\text{material})}^{2-}\;\;+\;{\text{CO}}_{2(\text{gas})}\;+\;{\text{H}}_{2}{\text{O}}_{\left(\text{gas}\right)}\;↔\;2{\text{HCO}}_{3}^{-} \end{array} \end{array}$$

When tests are performed in humid air, both reactions take place, while in the case of dry air only the first one happens. These reactions explain the greater response when the tests are carried out in humid environment.

### 3.4. Repeatability of the Sensors Performance

**Figure 7.**
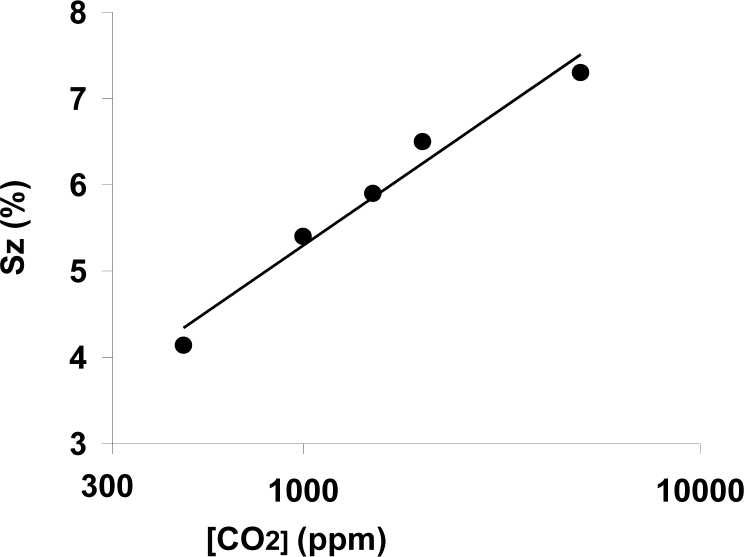
Sensitivity to carbon dioxide within a range 500 to 5,000 ppm, measured at 300 °C and 40% RH in terms of impedance change.

After confirming the coincidence of the prototype sensitivity and response time with the results obtained in earlier experiments with externally heated laboratory samples, the subsequent experiments were carried out using the signal conditioning circuit designed, thus, the impedance module (Z) was registered. The sensitivity to carbon dioxide in terms of the impedance module is shown in Figure 7 is for the same concentration ranges previously shown in Figure 5.

The first tests performed in relation to the repeatability of the sensors performance aimed to test the repeatability of the fabrication process, by testing sensors fabricated in different batches. For this reason, four different batches were fabricated within six months. The initial impedance of the samples was measured on the four different batches, and a dispersion of 20% was observed.

**Figure 8.**
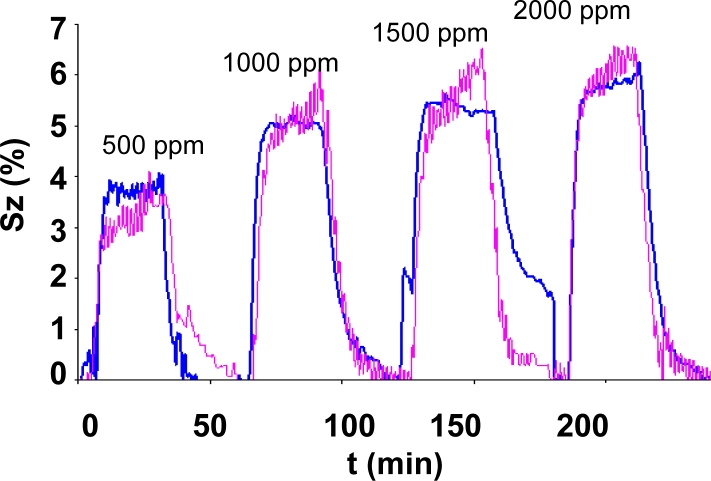
Dynamic response to carbon dioxide of two different samples within a range of 500 to 2,000 ppm, measured at 300 °C and 40% RH.

Regardless of the dispersion of the impedance, the dynamic response was measured on all four batches and its repeatability confirmed. As an example, Figure 8 shows the comparison of the response of two sensors fabricated in two different batches. A maximum difference of 8% in the response of the values was recorded in the tests performed to sensors of all the batches.

**Figure 9.**
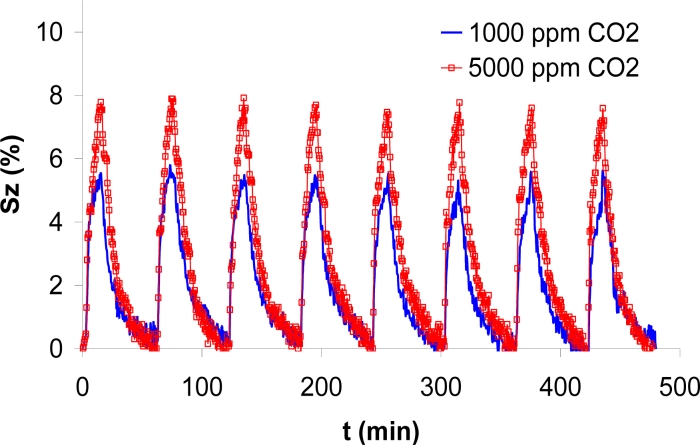
Comparison of eight consecutive pulses of two different concentrations of CO_2_, measured at 300 °C and 40% RH.

The following tests were performed by repeating eight times pulses of 15 minutes in carbon dioxide, followed by 45 minutes in clean air, in tests that lasted a total of eight hours. Those tests were repeated for different concentrations. As an example, the response to 1,000 ppm and to 5,000 ppm is shown in Figure 9. The first graph (1,000 ppm) was obtained after testing the prototype during 3 days, and the second one (5,000 ppm) was obtained after nine days of operation. As it can be observed, the repeatability of the pulses is good, having a maximum difference of 7% between different measurements.

**Figure 10.**
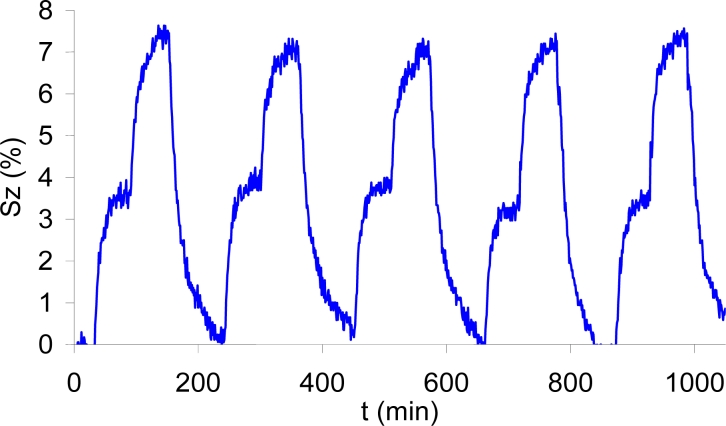
Eight consecutive pulses of 400+5,000 ppm, measured at 300 °C and 40% RH.

The following test was performed applying pulses of gas from 400 to 5,000 ppm (Figure 10). The aim of this test was to check the variation of the sensor response starting from the usual background concentration outdoors (around 400 ppm) and changing to another concentration. The results show that also in this case the repeatability of the pulses is in the same range as in the tests before. These results were obtained after five days of continuous operation of the sample.

### 3.5. Cross-Sensitivity to Interfering Gases

Regarding the issue of cross-sensitivity, two gases of relevance in Indoor Air Quality (IAQ) applications were tested: methane (CH_4_) and carbon monoxide (CO). Methane is the main component of natural gas, which is flammable and explosive. The lower explosive limit (LEL) of this gas is 5% and the standards in Europe require the detection of the 20% of the LEL (which is 1% of CH_4_). This was the concentration tested. In relation to carbon monoxide, this gas is originated in the partial combustion of any fossil fuel and it is very poisonous at even low concentrations. For example, OSHA’s limit for 8 hour exposure is set to 50 ppm. Tests were performed in this case from 5 to 100 ppm.

**Figure 11.**
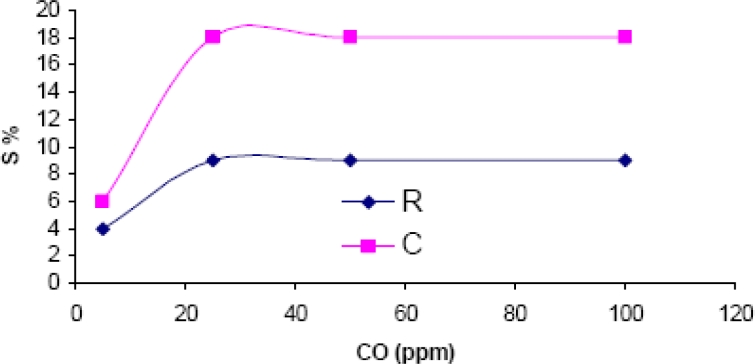
Response to carbon monoxide measured at 300 °C and 40% RH.

The results showed no response to methane in the same test conditions used for the former experiments (40% relative humidity and 300 °C operating temperature). When response to carbon dioxide was tested (Figure 11), it could be seen that saturation was reached over 20 ppm CO. The response magnitude was similar to the response to carbon dioxide, but the response time was 12 minutes, thus, much longer than for carbon dioxide (3 minutes). This difference in the velocity of response can be use to discriminate one gas from the other.

## 4. Conclusions

A prototype with an integrated Pt heater was fabricated and tested to check some aspects of its performance as a carbon dioxide sensor in air quality applications. The uniformity of the temperature on the sensing layer was confirmed by the thermographic camera results. Humidity influence on the sensor performance was been tested, and it was confirmed that between 40 and 80% relative humidity its influence on the sensitivity of the layer is almost negligible. The repeatability of the fabrication process was checked within four different batches, showing that the dynamic response of the fabricated prototypes is the same in samples fabricated in different sets. The repeatability of the response of the sensors was checked by submitting a sample to several repeatability tests within 30 days with satisfactory results, with dispersion in the responses always below 8%. Regarding cross-sensitivity, the sensor does not respond to CH_4_ at all, and although it responds to CO, this gas could be discriminated through the difference in the velocity of response, which is four times slower. All the obtained results corroborate the suitability of a BaTiO_3_-CuO sputtered thin film for carbon dioxide detection in air quality applications.

## Figures and Tables

**Table 1.** Some electrochemical and NDIR sensors available for carbon dioxide detection [1–3].

**Sensor type**	**Company**	**Model**	**Range of Detection**	**Price (approx)**
Electrochemical	Figaro	TGS4161 TGS4160	350–10,000 ppm 350–50,000 ppm	20 €
	Alphasense	CO2-D1	0.2–95%	30 €
	Hanwei Electronics	MG811	350–10,000 ppm	15 €
NDIR	Alphasense	IRC-A1	0–5,000 ppm (min) 0–100% (max)	75 €
	Dynament	TDS003	0–1000 ppm (min) 0–50% (max)	85 €
		TDS0048	0–500 ppm (min) 0–100% (max)	130 €
